# Creativity supports learning through associative thinking

**DOI:** 10.1038/s41539-025-00334-1

**Published:** 2025-07-01

**Authors:** Simone A. Luchini, James C. Kaufman, Benjamin Goecke, Oliver Wilhelm, Yoed N. Kenett, Daisy Lei, Mathias Benedek, Janet G. van Hell, Roger E. Beaty

**Affiliations:** 1 Department of Psychology, Pennsylvania State University, Philadelphia, USA PA; 2 Neag School of Education, University of Connecticut, Storrs, USA CT; 3 Hector Research Institute of Education Sciences and Psychology, University of Tübingen, Tübingen, Germany; 4 Institute of Psychology and Education, Ulm University, Ulm, Germany; 5 Faculty of Data and Decision Sciences, Technion—Israel Institute of Technology, Haifa, Israel; 6 Department of Psychology, University of Graz, Graz, Austria

**Keywords:** Psychology, Human behaviour

## Abstract

Creativity is a key 21st-century skill and a consistent predictor of academic learning outcomes. Despite decades of research on creativity and learning, little is known about the cognitive mechanisms underlying their relationship. In two studies, we examined whether creativity supports associative learning through associative thinking—the ability to generate novel word associations—an ability central to creativity which has not been previously tied to associative learning. In Study 1, we found that students who generated more novel word associations learned more words on a foreign language learning test 24 h later. In Study 2, we replicated and extended the effect to naturalistic creativity tasks (i.e., writing short stories and sketching line drawings), finding associative thinking mediated the relationship between creativity and associative learning. Importantly, both studies controlled for general intelligence. Our findings suggest that creativity’s contribution to learning operates partly through a shared cognitive capacity for making new connections.

## Introduction

In today’s complex world, creativity is essential for academic and career success. Creativity consistently predicts learning outcomes, with robust links between creative abilities and academic achievement^1–4^. How does creativity contribute to learning? In the present research, we explore one cognitive process that may tie creativity and learning: *associative thinking*, the ability to make connections between concepts. Creativity involves connecting pieces of information to form ideas; likewise, learning, particularly associative learning (e.g., foreign language vocabulary learning), involves linking new information to existing concepts in memory. However, whether associative thinking directly enhances learning remains unknown. We conducted two studies with undergraduate students to assess the role of associative thinking in paired-associate language learning, testing whether associative thinking can explain the relationship between creativity and learning.

Creativity and learning are multifaceted constructs that have been studied in a variety of ways. Here, we operationalize creativity as divergent thinking—the ability to produce diverse solutions to open-ended problems^5^. One of the cognitive processes long considered important for creativity is associative thinking^6–8^. Creativity utilizes both free association (i.e., spontaneously connecting concepts through bottom-up memory activation) and goal-directed association (i.e., strategically combining concepts through top-down control). Although associative thinking resembles key learning processes—particularly associative learning—no study has tested its role as a shared mechanism of learning and creativity.

Educational research on learning and creativity has mainly focused on correlating outcome variables, such as GPA and divergent thinking performance. A meta-analysis found that creativity predicts academic achievement (*r* = 0.22), indicating a small but significant positive relationship between academic accomplishment—often considered a marker of learning—and creativity^1^. Subsequent work suggests relations between academic learning and creative ability may be domain-specific, with domain knowledge providing a necessary condition for creativity in a domain (e.g., math and language)^9^. These findings highlight creativity’s contribution to important learning outcomes—pointing to potential domain-specific effects—though they say less about how creativity and learning relate at a mechanistic level. Further, such studies did not account for the confounding role of intelligence, which has independently been linked to learning^10,11^, academic achievement^12,13^, and creativity^14^.

Research in the field of intelligence has viewed learning and creativity as related cognitive abilities. In particular, divergent thinking has been linked to the broad cognitive ability of long-term storage and retrieval, known as Glr^15^. Glr consists of two facets: 1) encoding information into long-term memory (learning efficiency; Gl) and 2) fluently retrieving it (broad retrieval ability; Gr)^16^. This splitting of Glr is supported by evidence that both Gr and Gl can be separated psychometrically^17^. The vast majority of research examining the relationship between Glr and divergent thinking has focused on broad retrieval ability examined via fluency tasks (e.g., listing as many items from a given category as possible), showing that people who perform better on fluency tasks generate more (and more original) ideas on divergent thinking tasks^18–21^. Notably, broad retrieval ability is commonly assessed with tasks of ideational fluency—efficiently producing conceptually related words, which is a form of associative thinking^22,23^. However, this work has only linked performance on broad retrieval ability and divergent thinking tasks, rather than examining how associative thinking may support learning in relation to creativity.

In addition to intelligence, personality traits related to creativity may also facilitate learning through associative thinking. Openness to experience—a personality factor that consistently predicts creative performance^24–26^—supports creativity in part through larger vocabulary knowledge^27^ (i.e., knowing more words) as well as more “flexible” associative networks in semantic memory^28^ (i.e., organizing words in a more efficient way). People who are higher on openness have shown superior associative thinking, generating more semantically distant word associations^29^. Regarding learning, research found openness predicted performance on paired associate learning tasks, which involve forming arbitrary connections between unrelated items or words^30^ (e.g. learning to associate the word “house” with “flower”). The ability to make these types of associations may help highly open/creative individuals link new or unknown concepts to their existing knowledge. Indeed, flexible associative networks in semantic memory like those associated with high openness were also found to predict successful learning^31^. Given the established link between openness and creative performance, this research affords preliminary evidence that creativity may contribute to associative learning.

To our knowledge, only one study has assessed the relationship between creativity and learning using naturalistic measures of creative performance (e.g., writing and sketching)^18^. In this study, learning was assessed using paired associate learning tests, such as learning nonsense names for pictures and recalling them after a brief delay. Creativity was assessed by poem writing and drawing tasks, which were rated for creativity. Results showed that recall correlated significantly with rated creativity, suggesting that people who learn paired associations more effectively also produce more original ideas. Although this study established a link between associative learning and creativity, whether associative thinking plays a role in explaining this relationship remains unclear.

Creativity is a consistent predictor of learning outcomes like academic achievement^1,3,4^. Yet despite decades of research, how creativity and learning relate at a fundamental cognitive level remains poorly understood. In the present research, we investigate whether associative thinking may be a shared cognitive mechanism driving the link between creativity and learning.

We conducted two studies with undergraduate students to examine the role of associative thinking in paired-associate language learning, using the Learning Efficiency Test (LET). The LET assesses how quickly and durably people acquire new verbal information using a foreign language learning paradigm. This paradigm involves learning Lithuanian-English word pairs^32^. Lithuanian was chosen because its relative obscurity for English speakers controls for facilitative effects of prior knowledge and exposure. To assess associative thinking, we used a verb generation task that requires generating creative/unusual word associations. The verb generation task has been used in several studies to investigate associative thinking in the context of semantic memory^33,28,34–36^. Word associations were computationally scored for semantic distance using distributional semantic models^37^, providing an objective measure of novelty.

In Study 1, we tested whether students who generated more semantically distant word associations also learned more words on the LET when assessed during the study session and then 24 h later. In Study 2, we assessed creative performance on naturalistic tasks of writing stories and drawing sketches. We tested whether associative thinking mediates the relationship between creative performance and learning, examining it as a potential shared cognitive mechanism. We also distinguished between free association and goal-directed association to determine if any relationship between associative thinking and learning is driven by spontaneous vs. controlled association. We thus examined whether the ability to make connections between disparate concepts is fundamental to both creativity and learning.

## Results

### Study 1

In Study 1, we tested whether associative thinking supports associative learning. To assess associative thinking, participants completed a verb generation task, which presented nouns (e.g., *pencil*) and required generating verbs that could be creatively associated with them (e.g., *shade*). This word association task measures goal-directed association—participants had the goal of producing a creative association that was novel yet meaningful. Goal-directed association differs from free association, where people say the first word that comes to mind^6^. Participants also completed the LET to assess paired-associate learning, as well as intelligence tasks to control for general cognitive ability. We hypothesized that the ability to generate creative, goal-directed associations (assessed by the semantic distance of responses) would predict paired-associate learning performance on the LET (assessed both during and 24 h after the session).

### Comparisons, correlations and measurement model

We computed descriptive statistics for day 1 learning (words recalled on the final day 1 test with no feedback) and day 2 learning (words recalled on day 2 with no feedback). As expected, a paired-samples t-test revealed that day 1 learning was significantly higher (*M* = 19.64, *SD* = 11.23) than day 2 (*M* = 16.34, *SD* = 10.21), *t*(141) = 7.51, *p* < 0.001, *d* = 0.31.

**Fig. 1:**
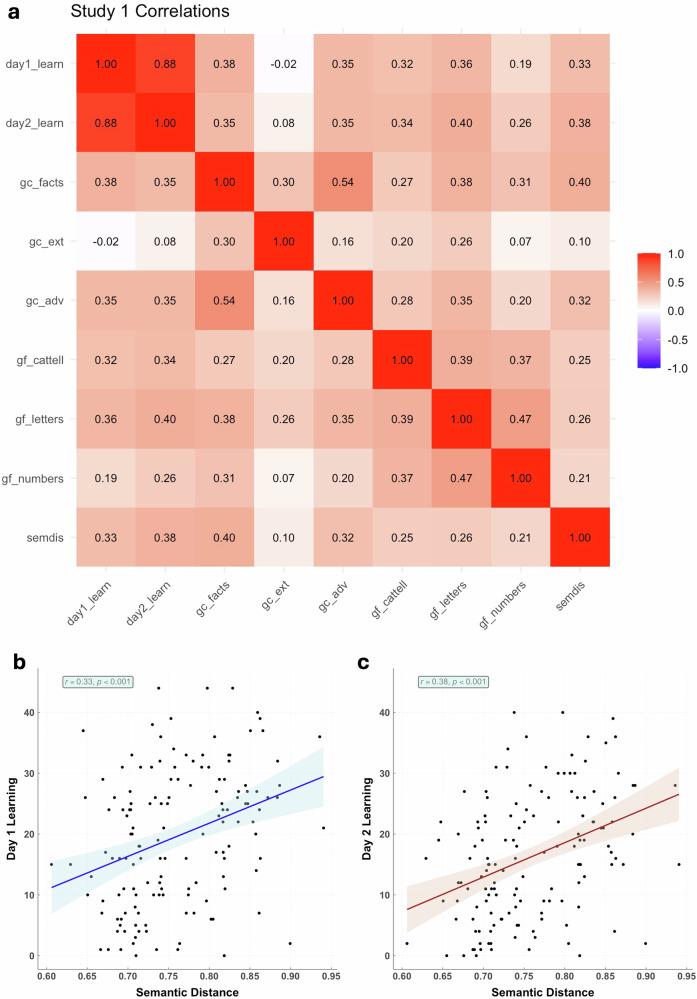
Peason correlations between all observed variables in study 1. Pearson correlations between (**a**) our observed variables, (**b**) day 1 learning and semantic distance on the verb generation task (Semdis), and (**c**) day 2 learning and Semdis. *r* > 0.15 is associated with *p* < 0.05. **a** day1_learn = number of correctly recalled LET words on day 1; day2_learn = number of correctly recalled LET words on day 2; gc_facts = crystallized intelligence, factual knowledge test; gc_ext = crystallized intelligence, extended range tests; gc_adv = crystallized intelligence, advanced vocabulary knowledge; gf_cattell = fluid intelligence, series completion task; gf_letters = fluid intelligence, letter sets task; gf_numbers = fluid intelligence, number series task; semdis = mean semantic distance values for verb generation task.

Pearson correlations were then computed and are visualized with a heatmap in Fig. 1. As expected, day 1 learning showed a large correlation with day 2 learning (*r* = 0.88, *p* < 0.001). More importantly, associative thinking (semdis) possessed medium correlations with both day 1 learning (*r* = 0.33; *p* < 0.001) and day 2 learning (*r* = 0.38; *p* < 0.001): people who produced more semantically-distant word associations recalled more words learned on day 1 and better retained them 24 h later.

**Fig. 2:**
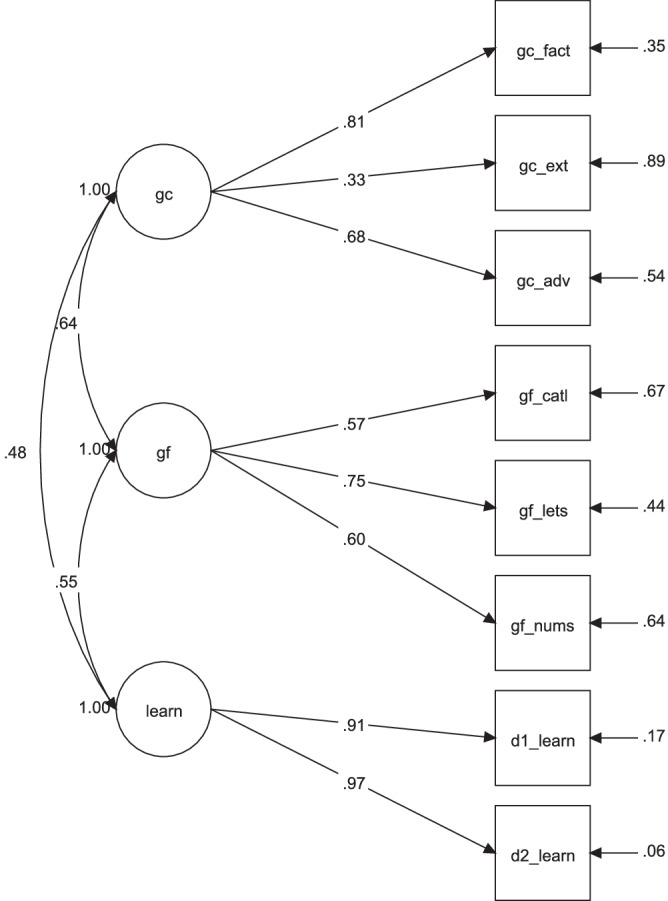
Confirmatory factor analysis of two intelligence variables and word learning. Description of the measurement model in study 1, testing the relationships between intelligence measures and learning. gc = crystallized intelligence; gf = fluid intelligence; semdis = semantic distance. d1_learn = number of correctly recalled LET words on day 1; d2_learn = number of correctly recalled LET words on day 2; gc_fact = crystallized intelligence, factual knowledge test; gc_ext = crystallized intelligence, extended range tests; gc_adv = crystallized intelligence, advanced vocabulary knowledge; gf_catl = fluid intelligence, series completion task; gf_lets = fluid intelligence, letter sets task; gf_nums = fluid intelligence, number series task.

Regarding intelligence, we specified a measurement model for the two facets (fluid and crystallized; *Gf* and *Gc*) and learning (day 1 and day 2; Fig. 2). This model fit the data well: χ2 (17) = 24.009, *p* = 0.119; CFI 0.981; RMSEA = 0.053 [90% CI: 0.000, 0.099]; SRMR = 0.045. As expected, factor scores for *Gf* and *Gc* were strongly correlated, *r* = 0.774, *p* < 0.001, yet psychometrically distinct from one another. Regarding factor scores of learning, we found medium to large correlations with *Gc* (*r* = 0.559, *p* < 0.001) and *Gf* (*r* = .638, *p* < 0.001), consistent with past work suggesting considerable common variance shared between general cognitive abilities and word learning.

### Regression models: predictors of learning

Next, we specified two regression models to assess contributions of intelligence facets and associative thinking to word learning. Our first model focused on intelligence facets (*Gf* and *Gc*) predicting word learning (day 1 and day 2 combined factor), using the measurement model above, finding a significant effect of *Gf* (*β* = 0.405, *p* = 0.004) but not *Gc* (*β* = 0.224, *p* = 0.146).

**Fig. 3:**
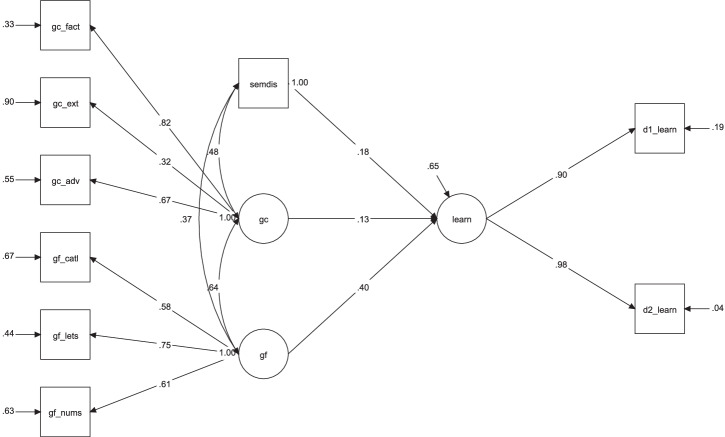
Regression model testing the effect of associative thinking on learning beyond intelligence variables. Description of the second regression model in study 1, testing whether associative thinking predicts learning beyond intelligence variables. gc_fact = crystallized intelligence, factual knowledge test; gc_ext = crystallized intelligence, extended range tests; gc_adv = crystallized intelligence, advanced vocabulary knowledge; gf_catl = fluid intelligence, series completion task; gf_lets = fluid intelligence, letter sets task; gf_nums = fluid intelligence, number series task; semdis = mean semantic distance for verb generation task; d1_learn = number of correctly recalled LET words on day 1; d2_learn = number of correctly recalled LET words on day 2.

We then added associative thinking (a single manifest variable) to the regression model, testing the critical question of whether associative thinking predicts learning beyond intelligence, χ² (22) = 25.290, *p* = 0.283; CFI = 0.991; RMSEA = 0.032 [90% CI: 0.000, 0.079]; SRMR = 0.042 (Fig. 3). Although *Gf* strongly predicted learning (*β* = 0.399, *p* = 0.002), semantic distance showed a significant (albeit small) effect on learning (*β* = 0.182, *p* = 0.037); the effect of *Gc* on learning remained non-significant (*β* = 0.130, *p* = 0.367), despite the relation between *Gc* and semantic distance (*β* = 0.478, *p* < 0.001). Taken together, the results demonstrate that associative thinking predicts learning beyond general cognitive ability.

### Study 2

Study 1 provided the first evidence that associative thinking predicts learning in the context of a foreign language learning test. Students who generated more semantically distant word associations performed better on the paired associate learning test, and this effect remained significant beyond general cognitive ability. While the correlations between associative thinking and learning were strong, the unique effect controlling for intelligence was more modest, yet remarkably still detectable given that associative thinking was measured with a single task compared to the multi-task latent variables used for fluid and crystallized intelligence. Notably, while fluid intelligence remained a strong predictor, crystallized intelligence was no longer significant when controlling for associative thinking, suggesting some shared variance between these constructs.

One way that creativity may support learning is through associative thinking: the ability to make new connections between concepts. In Study 2, we sought to test this hypothesis by investigating whether creativity is associated with learning and whether associative thinking mediates any such relationship. We measured creativity via naturalistic tests by assessing creative performance on tasks involving writing stories and drawing sketches. We also looked to replicate and extend findings from Study 1, by distinguishing between free association and goal-directed association to determine if the relationship between associative thinking and learning is driven by spontaneous association (producing the first word that comes to mind, regardless of novelty) or goal-directed association (intentionally producing a novel association). This distinction allows us to model goal-directed associative ability while controlling for baseline associative processes. Critically, we tested whether associative thinking mediates the relationship between creative performance and learning, examining it as a potential shared cognitive mechanism.

Creativity is a higher-order construct which engages many lower-order, functionally distinct processes like attention and memory^38,39^. Associative thinking is one such form of lower-order cognition that supports creativity by driving the connection of distant concepts in memory^6^. Testing if associative thinking mediates the effect of creativity on learning thus evaluates whether any of the remaining lower-order processes of creativity, over and above associative thinking, are driving this link. This test of mediation is thus justified by the hierarchical perspective which places associative thinking as a component of creativity, and not vice versa. Further, this structural organization is informed by past work which linked creativity with learning, while leaving the role of associative thinking unexplored^18^.

### Learning performance across conditions

We first calculated descriptive statistics for day 1 and day 2 learning. Replicating Study 1, we found that day 1 learning was higher (*M* = 20.5, *SD* = 11.6) than day 2 (*M* = 17.54, *SD* = 11.25), *t*(145) = 7.58, *p* < 0.001, *d* = 0.25. We also found a large positive correlation between day 1 and day 2 learning, consistent with Study 1 (*r* = 0.92, *p* < 0.001).

**Fig. 4:**
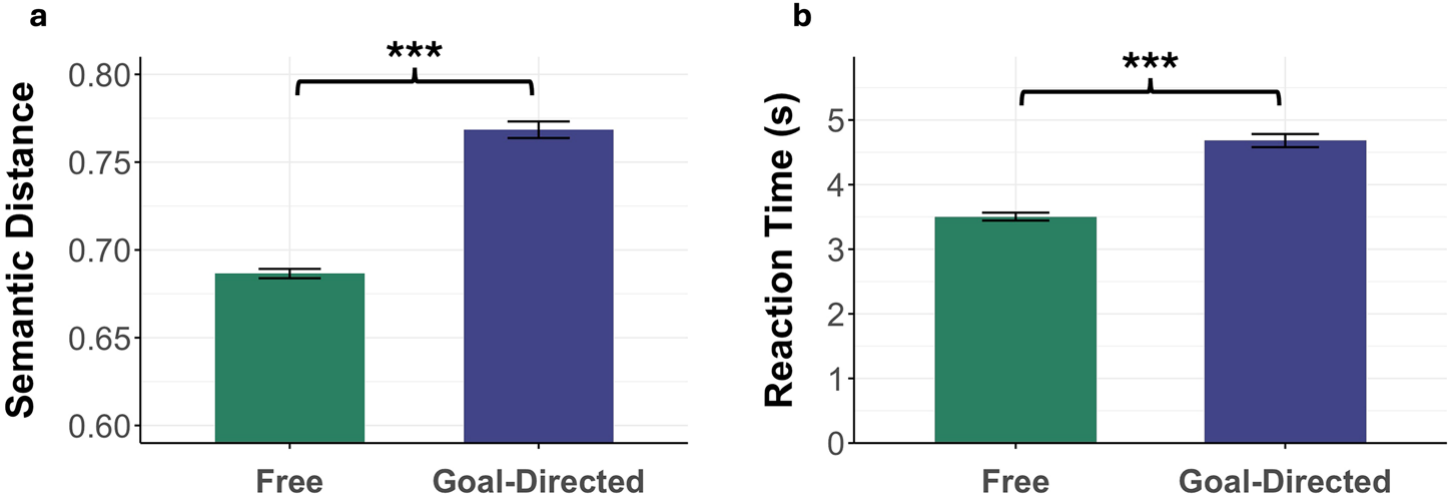
Performance measures on the verb generation task across free and goal-directed conditions. Comparison of free and goal-directed association on the verb generation task in terms of (**a**) mean semantic distance and (**b**) mean reaction time in seconds. Error bars represent the standard error. *** = *p* < 0.001.

Next, we ran a series of paired samples t-test analyses to test differences in performance across conditions of the verb generation test, as well as item type. We compared the semantic distance between free and goal-directed associations. As expected, semantic distance was higher for goal-directed (*M* = 0.77, *SD* = 0.06) than for free (*M* = 0.69, *SD* = 0.03) association, *t*(142) = 17.79, *p* < 0.001, *d* = 1.0 (Fig. 4). We also found that reaction times were faster during free association (*M* = 3.5 s, *SD* = 0.73) than goal-directed association (*M* = 4.69 s, *SD* = 1.2), *t*(142) = 12.43, *p* < 0.001, *d* = 1.14. These results replicate past work^29^ and indicate that it takes participants more time to generate novel word associations, and these associations are more semantically distant than free associations.

We then explored whether using previously studied nouns (from the LET) on the verb generation task impacted the semantic distance of associations (compared to non-studied nouns). This analysis revealed that the prompt type (studied vs. non-studied) impacted goal-directed association, with non-studied nouns (*M* = 0.78, *SD* = 0.06) displaying higher semantic distance than LET nouns (*M* = 0.76, *SD* = 0.06), *t*(142) = 4.2, *p* < 0.001, *d* = 0.25, with no effect on free association. This exploratory analysis suggests that the prior study of nouns may have interfered with the ability to generate novel word associations, without affecting free association.

### Correlations between learning, associative thinking, and creativity

**Fig. 5:**
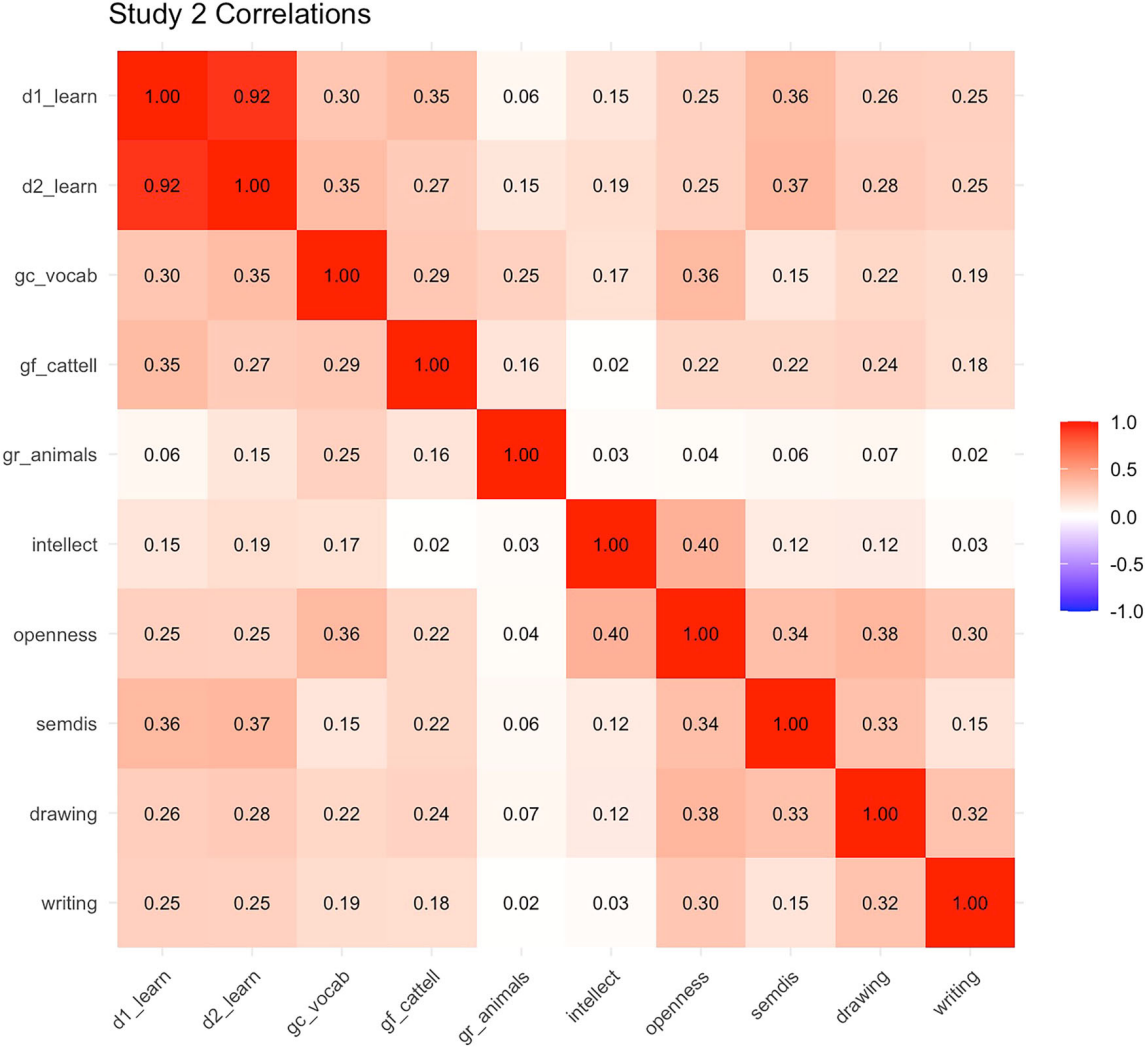
Pearson correlations between all observed variables in study 2. Results from pairwise Pearson correlations ran between all observed variables in study 2. *r* > 0.16 is associated with *p* < 0.05. d1_learn = number of correctly recalled LET words on day 1; d2_learn = number of correctly recalled LET words on day 2; gc_vocab = crystallized intelligence, advanced vocabulary knowledge; gf_cattell = fluid intelligence, series completion task; gr_animals = broad retrieval ability, animal fluency task; intellect = BFAS intellect; openness = BFAS openness; semdis = mean semantic distance values for verb generation task; drawing = AuDrA average scores from 7 drawings; writing = DSI average scores from 3 short stories.

Next, we computed Pearson correlations between day 2 learning, associative thinking, and creativity. Pearson correlations are visualized with a heatmap in Fig. 5. Note, we focus on day 2 learning here, as this provides a stricter measure of learning, but results are comparable for day 1.

We assessed whether day 2 recall of learned words relates differentially to free vs goal-directed association, measured through semantic distance. Although the two were positively correlated (*r* = 0.34, *p* < 0.001), only goal-directed association showed a significant correlation with day 2 learning (*r* = 0.23, *p* = 0.005); while free association showed a non-significant correlation (*r* = 0.01, *p* = 0.89). These findings indicate that the associative thinking-learning effect is related to the ability to deliberately (rather than spontaneously) combine semantically distant concepts. We then computed a difference score (goal-directed – free association) for semantic distance on the verb generation task, treating the free association condition as a baseline and allowing us to use the full verb generation dataset in subsequent analyses. As expected, this difference score showed a medium positive correlation with day 2 learning (*r* = 0.37, *p* < 0.001).

We also tested how day 2 learning relates to creativity and personality measures. Notably, drawing and writing were positively correlated but distinct (*r* = 0.32, *p* < 0.001). We found that creative writing ability, measured as average DSI originality scores of all short stories, predicted day 2 learning (*r* = 0.25, *p* = 0.002). Similarly, drawing creativity, measured as the average AuDrA originality scores of all drawings, was also positively correlated with day 2 learning (*r* = 0.28, *p* = 0.001). Regarding personality, we found both Openness (*r* = 0.25, *p* = 0.003) and Intellect (*r* = 0.19, *p* = 0.03) related to day 2 learning.

Overall, the results of Study 2 replicate Study 1’s findings on associative thinking and paired-associate learning—specifically linking learning to goal-directed association (but not free association)—and extend them by showing that naturalistic creativity tasks (writing and drawing) and openness also predict learning.

### Regression models: predictors of learning

We ran a series of regression models using structural equation modeling. First, we specified a measurement model with a general intelligence factor (comprised of fluid and crystallized intelligence, and broad retrieval ability; *Gf*, *Gc* and *Gr*) and a learning factor (χ² (4) = 28.58, *p* < 0.001; CFI = 0.92; RMSEA = 0.21 [90% CI: 0.14, 0.28]; SRMR = 0.038). As expected, g showed a large positive effect on learning (β = 0.53, *p* < 0.001).

Next, we tested the relative contributions of associative thinking and intelligence to learning, using the semantic difference score for associative thinking (χ² (8) = 24.71, *p* = 0.002; CFI = 0.94; RMSEA = 0.12 [90% CI: 0.07, 0.18]; SRMR = 0.068). Results confirmed the associative thinking effect from Study 1: semantic distance (semdis) showed a medium effect on learning (*β* = 0.32, *p* < 0.001), accounting for the simultaneous effect of g on learning (*β* = 0.46, *p* < 0.001).

**Fig. 6:**
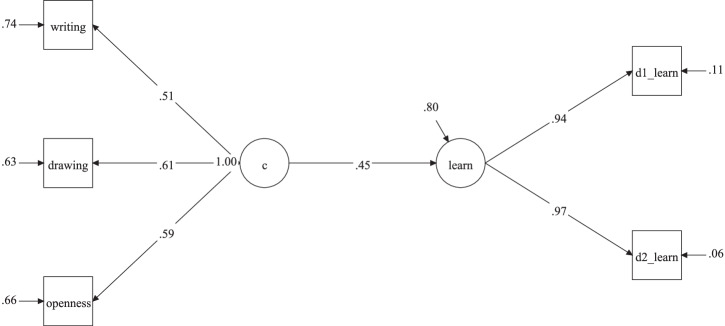
Regression analysis of a latent creativity factor on learning. Description of the third regression model in study 2, testing whether a latent creativity factor predicts learning.writing = DSI average scores from 3 short stories; drawing = AuDrA average scores from 7 drawings; openness = BFAS openness; d1_learn = Number of correctly recalled LET words on day 1; d2_learn = Number of correctly recalled LET words on day 2.

We then turned to creativity, assessing the extent to which a latent creativity factor (c)—indicated by drawing, writing, and openness—predicted retention (Fig. 6; χ² (4) = 0.85, *p* = 0.931; CFI = 1.00; RMSEA = 0.00 [90% CI: 0.00, 0.036]; SRMR = 0.010). As expected, this model yielded a medium effect of creativity on learning (*β* = 0.45, *p* < 0.001).

We then added associative thinking (semdis) to the regression analysis along with creativity (c), to test for unique effects of both variables (χ² (7) = 3.82, *p* = 0.800; CFI = 1.00; RMSEA = 0.00 [90% CI: 0.00, 0.07]; SRMR = 0.022). Despite creativity and associative thinking being positively correlated (*r* = 0.49), this model yielded a unique effect of creativity (*β* = 0.39, *p* = 0.005) on learning, as well as a (notably reduced) small effect of associative thinking (*β* = 0.24, *p* = 0.047), indicating partially distinct contributions of creativity and associative thinking to learning.

### Mediation analysis: role of associative thinking

**Fig. 7:**
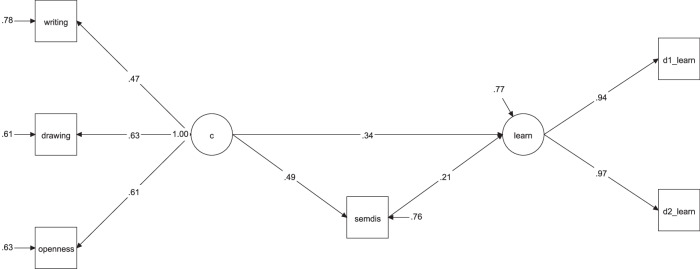
Mediation model showing creativity predicting learning through associative thinking. Description of the first mediation model in study 2, testing whether associative thinking mediates the relationship between a latent creativity factor and learning. writing = DSI average scores from 3 short stories; drawing = AuDrA average scores from 7 drawings; openness = BFAS openness; semdis = difference score between semantic distance on free and goal-directed verb generation task; d1_learn = Number of correctly recalled LET words on day 1; d2_learn = Number of correctly recalled LET words on day 2.

To address our core question, we conducted a mediation model to determine whether associative thinking (semantic distance) mediates the relationship between creativity and learning (Fig. 7; χ² (7) = 3.82, *p* = 0.800; CFI = 1.00; RMSEA = 0.00 [90% CI: 0.00, 0.07]; SRMR = 0.022). Critically, the results indicated a partial mediation: creativity had a significant effect on associative thinking (β = 0.49, *p* < 0.001), which in turn significantly predicted learning (*β* = 0.21, *p* = 0.045). The direct effect of creativity on learning was also significant (*β* = 0.33, *p* = 0.005), which supports a partial mediation effect. Altogether, our findings point to associative thinking as a cognitive mechanism linking creativity and learning.

**Fig. 8:**
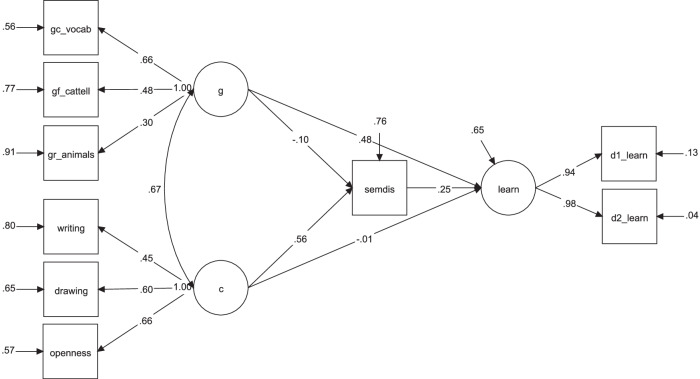
Mediation model showing creativity predicting learning through associative thinking when accounting for general intelligence. Description of the second mediation model in study 2, testing whether associative thinking mediates the relationship between a latent creativity factor and learning, beyond intelligence. gc_vocabulary = crystallized intelligence, advanced vocabulary knowledge; gf_cattell = fluid intelligence, series completion task; gr_animals = broad retrieval ability, animal fluency task; writing = DSI average scores from 3 short stories; drawing = AuDrA average scores from 7 drawings; openness = BFAS openness; semdis = difference score between semantic distance on free and goal-directed verb generation task; d1_learn = Number of correctly recalled LET words on day 1; d2_learn = Number of correctly recalled LET words on day 2. *N* = 145.

Finally, we ran an additional model including intelligence as a covariate (χ² (22) = 30.22, *p* = 0.113; CFI = 0.98; RMSEA = 0.051 [90% CI: 0.00, 0.09]; SRMR = 0.037; Fig. 8). While the previous model showed partial mediation, adding intelligence revealed full mediation: both direct effects of intelligence (*β* = 0.480, *p* = 0.072) and creativity (*β* = −0.015, *p* = 0.957) on learning were non-significant, while the indirect path from creativity through associative thinking remained significant (creativity → associative thinking: *β* = 0.558, *p* = 0.006; associative thinking → learning: *β* = 0.250, *p* = 0.020). Of note, the direct effect of intelligence was only marginally non-significant, indicating that a larger sample size could have revealed a significant effect. Altogether, our findings suggest that controlling for intelligence accounts for the remaining direct effect of creativity on learning, supporting associative thinking as the primary cognitive mechanism linking creativity to learning outcomes.

## Discussion

The relationship between creativity and learning is well established, but the cognitive mechanisms underlying this link have remained poorly understood. Across two studies, we investigated whether associative thinking—the ability to form novel connections between concepts—serves as a shared cognitive mechanism driving the link between creativity and learning. Study 1 established a relationship between associative thinking and learning, finding that the ability to generate semantically distant word associations predicted vocabulary recall immediately after learning and vocabulary retention after a 24 h delay. Study 2 replicated and extended this associative thinking-learning relationship, critically showing that 1) goal-directed association, more so than free association, explains the correlation with learning, and 2) associative thinking mediates the relationship between creativity and learning. Our findings suggest that creativity contributes to learning in part through a shared capacity for forming new connections.

Importantly, in both studies, associative thinking remained a robust predictor of learning even after simultaneously considering general intelligence. In Study 1, both fluid and crystallized intelligence correlated with paired-associate learning, as expected. However, structural equation modeling revealed that only fluid intelligence and associative thinking remained significant predictors of learning, while crystallized intelligence (vocabulary knowledge) did not. This finding is particularly notable: despite the well-established link between vocabulary knowledge and vocabulary learning^40,41^, associative thinking emerged as a stronger predictor than vocabulary knowledge when both were considered simultaneously in a structural equation model. These results suggest that the ability to form distant associations between existing concepts may be more crucial for learning than merely possessing a larger vocabulary size.

Our findings on individual differences in associative thinking complement research on memory processes in paired associate learning. Although some work has focused on how specific strategies like elaboration support the transfer of information from working memory to long-term memory^42^, our results suggest that stable individual differences in associative thinking ability may provide domain-general support for learning new associations. Just as elaborative encoding can strengthen memory formation during the learning process, having a greater capacity to form novel semantic connections may facilitate the initial binding of new associations. This suggests multiple routes to successful learning—both through the strategic deployment of memory processes and through individual differences in core cognitive abilities that support associative thinking. Future research could examine potential interactions between these factors, investigating whether individuals with stronger associative thinking abilities are also more likely to spontaneously employ effective encoding strategies.

Regarding Study 2, we found that the effect of associative thinking on learning was driven by goal-directed association—the ability to generate creative associations that are original and meaningful^6,29^. In contrast, free association—producing the first related word that comes to mind—showed a non-significant correlation with learning, despite being highly correlated with goal-directed association. This distinction between goal-directed and free association is consistent with the effortful process of associative learning, which requires deliberately establishing connections between new items and existing knowledge. Our findings align with previous research that found goal-directed association on the verb association task, i.e., cuing people to think creatively when forming associations, yielded stronger correlations to intelligence and creativity compared to free word association^29^. Our work further establishes goal-directed association as an important cognitive ability, with predictive power beyond general intelligence.

Study 2 also explored whether prior learning of associations between known and foreign words (on day 1) would influence performance on the verb generation task (on day 2), aiming to understand how recently acquired knowledge might affect associative thinking. Specifically, we examined whether using previously studied nouns from the LET would impact the semantic distance of word associations compared to non-studied nouns. Interestingly, we found that goal-directed associations were significantly less semantically distant for studied nouns, suggesting that prior learned associations may interfere with the ability to generate novel connections^43^. This finding is in line with prior work indicating that increased knowledge of a concept leads to a reduction in the originality of related ideas, likely due to interference from conceptually related information^44^. Notably, this effect was not observed for free associations, indicating that interference arising from semantic richness only affects connections between distant concepts in memory. One possibility is that, during free association, participants are generating highly typical associations which are unaffected by the semantic richness of the noun. Future research could further investigate this apparent interference effect, exploring potential implications for creative tasks that require integrating new and existing knowledge across various domains.

Several studies have reported correlations between creativity and learning outcomes^1,9,45^, yet the cognitive mechanisms driving the creativity-learning relation have remained unclear. Study 2 provided mechanistic insight into this question: we found that associative thinking fully mediates the link between creativity (writing, drawing, and openness) and the retention of recently learned words. In this context, associative thinking involves connecting existing concepts, whereas learning involves connecting new items to existing knowledge. Thus, the ability to make connections between concepts—in a goal-directed manner—appears to be a shared cognitive mechanism underlying both creativity and learning.

Of note, our studies employed a single word learning paradigm to assess learning. Further work is required to determine if the present findings extend to non-language based learning (e.g., visual). It is possible that, given our reliance on verbal tasks to assess both learning and associative thinking, different effects may be observed for other task modalities. Further, the present studies could only establish a correlational link between creativity, associative thinking, and learning. In the future, causality should be investigated by experimentally testing whether enhancements of associative thinking, via training paradigms, can in turn benefit learning. Further research should also strive to map the shared neural processes of creativity, associative thinking, and learning as this would offer valuable insights into the mechanisms driving this relationship.

In addition, the extension of the present work to educational settings would provide a necessary degree of ecological validity. For instance, because our sample was composed entirely of university students enrolled in an introductory psychology course, it remains unclear whether the present findings will extend to students from other domains. Further work should also test whether the present findings extend to students of different ages, as past work has indicated a link between education, creativity, and semantic memory structures^46^.

The present data are correlational in nature. Although the modeling we use assigns causal roles such as explanans and explanandum to different variables, the design of the study is opaque with respect to the assignment of these roles. Therefore, competing and equivalent models can be specified that provide similarly good and sound accounts of the present data.

Taken together, our work clarifies the link between creativity and learning, establishing the role of associative thinking for the first time. We show that goal-directed associative thinking mediates the relationship between creativity and learning. Our findings open up potential avenues for interventions aimed at enhancing both creativity and learning^46,47^. As educators place greater emphasis on creativity, fostering associative thinking could be crucial for achieving positive learning outcomes.

## Methods

### Study 1

A total of 147 undergraduates from Pennsylvania State University completed both sessions of study 1. One participant was excluded for not following instructions, leaving a final sample of 146 (30 men; 113 women; 1 other/non-binary; 2 NA; mean age = 18.9 years, *SD* = 1.05 years). All participants were enrolled in an introductory psychology course and were recruited as part of a voluntary research-participation scheme. Students were given credits towards the completion of this introductory psychology course upon finishing the study. Informed consent was obtained from all participants, and the study received ethical approval from the Pennsylvania State University Institutional Review Board (00019378; Amy Sellers, Institutional Review Board Analyst).

### Procedure

The study was conducted over two days. On day 1, participants completed the LET^32^, which involves learning 45 Lithuanian-English word pairs. On day 2, approximately 24 h after finishing day 1, participants were re-tested on the Lithuanian-English word pairs. After the re-test, participants completed the verb generation test to measure associative thinking, a battery of intelligence tests measuring fluid intelligence (*Gf*) and crystallized intelligence (*Gc*), and other measures that were collected but not analyzed here. Both sessions were administered online and completed by participants on their personal computers.

### LET

Participants were first shown the 45 Lithuanian-English word pairs during an initial learning phase. Each trial visually presented a word pair for 4 s, preceded by a fixation cross lasting 1 s. Participants were instructed to memorize the English translation for each Lithuanian word, and were told in advance that they would later be tested. After this initial learning phase, participants took a test with immediate feedback. They were shown each Lithuanian word separately and given 4.5 s maximum to type the English translation. They could submit their response at any time within the allotted time frame. After submitting their response, the correct translation was displayed for 1.5 s as feedback.

This testing with immediate feedback was then repeated until the participant correctly recalled all word pairs. On each test iteration, correctly recalled words were dropped from the next round, gradually reducing the pool of word pairs to be recalled. Before each test run, participants also completed an unrelated distractor task in the form of 30 s of simple math problems. After solving each math problem, participants were provided feedback indicating whether their response was correct, which remained on the screen for 1 s. Once all word pairs were correctly recalled, participants passively restudied the entire 45 words like in the first run, completed 5 min of math problems, and took a final no-feedback test; the no-feedback test was repeated on day 2 before other tasks. While the original LET^32^ used Tetris for the final distractor round, we used math problems due to software/programming constraints. Although the LET was developed as a test of learning efficiency, we were only interested in word learning outcomes. As such, we only analyzed the number of correctly recalled words on the no-feedback tests as measures of day 1 and day 2 learning.

### Verb generation task

The verb generation task was designed to assess goal-directed associative thinking by instructing participants to generate remote associates^6,29^. Otherwise, the task broadly follows a typical word association paradigm. Each trial presented a noun, and participants generated a verb related to it. They were given instructions to think creatively and produce “a verb that is clearly related to the noun, but rarely used in association with the noun”. The noun remained on the screen for 10 s maximum, or until participants responded. A total of 20 nouns were selected from a larger pool from a previous study^29^.

Originality scores were derived by calculating semantic distance between each noun-verb association using the *SemDis* platform (semdis.wlu.psu.edu). Semantic distance represents the conceptual dissimilarity between two words in high-dimensional semantic space^48^. This metric correlates with human judgments of creativity^37,49^ and novelty^50,51^. To compute semantic distance, we used the global vectors model^52^ (i.e., GloVe), built by counting the co-occurrence frequencies in a 6 billion tokens corpus of Wikipedia and news texts. To model a latent variable, we created four indicators by 1) alphabetizing the cue words and 2) computing the mean semantic distance for five consecutive trials (e.g., belt, blade, boot, cafe, canoe). This approach allowed us to model error variance separately from true measurement variance^53^.

#### Gf

Three tests of Gf were included: the *series completion*, the *number series*, and the *letter sets task*. We selected the *series completion task* from the Culture Fair Intelligence Test (CFIT)^54^, which involved selecting the next image in a series of three progressively changing images from six options. Participants had 3 min to complete 13 matrices problems. The *number series task*^55^ required identifying the pattern in a sequence of numbers and selecting the next number that fit this pattern from five options. Participants had 5 min to complete 15 number series problems. The *letter sets task*^56^ (ETS Kit of Factor-Referenced Cognitive Tests) involved identifying which of five sets of four letters violated a common rule followed by the other four sets. Participants had 4 min to complete 15 letter sets problems.

#### Gc

We included three tests of Gc: the *advanced vocabulary*, *extended range*, and *factual knowledge tests*. The *advanced vocabulary* and *extended range tests* required selecting the word that more closely matched the meaning of a target word from four or five options^50^. Participants completed 25 trials from the advanced vocabulary test and 17 trials from the extended range test (8 min total). A *factual knowledge test*^57^ involved answering 32 general knowledge questions from a variety of topics (e.g., human physiology; American history) by selecting the correct response out of four options. Participants were given 10 min to complete this task.

### Study 2

We recruited 146 undergraduates from Pennsylvania State University; none had participated in Study 1. One participant was excluded for not following instructions, leading to a final sample of 145 for analysis (43 men; 101 women; 1 other/non-binary; mean age = 19.6, SD = 3.7). As with study 1, participants were recruited from a voluntary research-participation scheme as part of an introductory psychology course and were given credits towards the completion of this course upon finishing the study. Informed consent was obtained from all participants, and the study received ethical approval from the Pennsylvania State University Institutional Review Board (00019378 Amy Sellers, Institutional Review Board Analyst).

### Procedure

Study 2 also involved the LET and, just as Study 1, was conducted over the course of 2 consecutive days. We further included (in this order): the verb generation task (free- and goal-directed conditions), a story writing task, a drawing task, a matrix reasoning task (to assess *Gf*), the advanced vocabulary task (to assess *Gc*), an animal fluency task (to assess *Gr*), an openness scale, and other measures not analyzed here.

### Verb generation task

This task had two conditions: free and goal-directed. The goal-directed condition was the same as in Study 1; the free association condition involved a different set of instructions. Specifically, instead of being instructed to be creative in their associations, participants were only told to generate a verb “that is related to each noun.” To avoid any task interference effects, participants were always presented with the free version of the task before the goal-directed version^58^. Further, we collected reaction times for each trial, calculated as the time in seconds between the presentation of the cue word to when a response was submitted.

We increased the task to 40 items per condition (80 total), with 20 items selected from the LET (“studied”) and 20 from a related study^29^ (“non-studied’), matched by recall accuracy (for studied words) and concreteness (for non-studied words). The inclusion of both studied and non-studied words allowed us to explore whether associative thinking would vary based on prior exposure to the studied words. The contrast of studied and non-studied nouns was only relevant for this exploratory analysis. Like in Study 1, we computed semantic distance between each item and response pair using GloVe. A single semantic distance score was derived for each participant by averaging semantic distances across all items, both studied and non-studied. For each participant, we also calculated a difference score by subtracting the average semantic distance in the free condition from the goal-directed condition, providing a more sensitive metric of goal-directed association accounting for baseline semantic distance.

### Story writing task

The story writing task was included as a measure of creative writing skills. For each trial, participants were provided three words as prompts and instructed to write a short story around 5 sentences long^59,60^. Participants had 5 min to complete each trial and were further instructed to be creative and to use their imagination in coming up with the story. Participants completed three different trials including the prompts *year-week-embark*, *belief-faith-song*, and *stamp-send-letter*. Stories were evaluated by calculating divergent semantic integration (DSI)—a semantic-distance metric reflecting the integration of divergent ideas in text^59^. DSI is a natural language processing technique that calculates the cosine distance scores between all words in a text, which are then combined into a single composite score. DSI has been validated on a large dataset of responses to the story writing task, explaining up to 72% of the variance in human judgments of creativity^59^.

### Structured drawing task

The structured drawing task is a measure of figural creativity. Each trial of the structured drawing task involves the presentation of an incomplete shape, which participants are instructed to creatively incorporate into a sketch^61^. The task was administered via the online Crealyx platform (http://crealyx.com/). As participants completed the study on their personal computers, they were instructed to draw by using their mouse or trackpad. The creativity of drawings was automatically scored using automatic drawing assessment (AuDrA; https://osf.io/kqn9v/)^62^. AuDrA employs a convolutional neural network model that has been trained to predict the creativity ratings of thousands of sketches from the structured drawing task. Importantly, the model predictions have been shown to generalize to new sketches from the same task, explaining 64% of the variability in the human rated creativity scores^62^. Participants completed a total of 7 drawings, each involving a different incomplete shape as a cue. For each participant, a single figural creativity score was then derived by averaging AuDrA predictions across all drawings. All incomplete shapes in our structured drawing task were present in the training data of the AuDrA model, ensuring that the predictive strength of AuDrA would translate to the present dataset.

### Openness/intellect

We included the Openness and Intellect subscales from the Big Five Aspect Scale^63^ (BFAS). Openness and Intellect are the two factors that, together, make up the broader trait of Openness to Experience. Importantly, while related, the two factors have been found to measure distinct aspects of personality, and to possess their own unique relationships to intelligence^64^ and creativity^25,65^. Each of the two subscales involves 10 self-report items (e.g., “*I am intrigued by the patterns I find in art and nature*.”) which are rated on a 5-point Likert scale (Strongly Agree—Strongly Disagree).

### Animal fluency task

We administered the animal fluency task as a measure of Gr. Participants were instructed to generate as many animal names as possible in 1 min, and to continue working until the time ran out.

## Data Availability

Data has been made openly accessible: https://osf.io/rfjxs/?view_only=e772d03f07824b34960ca4f8d6006b12.

## Code availability

The codes supporting this work have been made openly accessible: https://osf.io/rfjxs/?view_only=e772d03f07824b34960ca4f8d6006b12.
